# Intraoperative Catastrophic Acute Pulmonary Embolism: A Case Report

**DOI:** 10.7759/cureus.59282

**Published:** 2024-04-29

**Authors:** Sara Gier, Jose L Diz Ferre, Sabry Ayad

**Affiliations:** 1 Outcomes Research, Ohio University Heritage College of Osteopathic Medicine, Cleveland, USA; 2 Outcomes Research, Cleveland Clinic Fairview Hospital, Cleveland, USA; 3 Outcomes Research, Cleveland Clinic, Cleveland, USA; 4 Anesthesiology, Cleveland Clinic, Cleveland, USA; 5 Anesthesiology, Cleveland Clinic Fairview Hospital, Cleveland, USA

**Keywords:** multidisciplinary care, veno-arterial extracorporeal membrane oxygenation (va ecmo), transesophageal echocardiogram, resuscitation, pulmonary embolism, biventricular failure, pulseless ventricular tachycardia, peripheral vascular disease, atrial fibrillation, bowel obstruction

## Abstract

This case report describes a 75-year-old female with a medical history including recurrent bowel obstruction due to sigmoid stricture, atrial fibrillation managed with rivaroxaban, a 50-year one pack-per-day smoking history, hypertension, hyperlipidemia, peripheral vascular disease with bilateral iliac stents (2015), stage III chronic kidney disease, and renal artery stenosis with bilateral stenting. She was transferred from outside hospital for an elective sigmoidectomy with ileorectal anastomosis following several recent admissions due to bowel obstruction that had been managed non-operatively. She was deemed optimized for surgery by the primary care team; however, during induction, she developed pulseless ventricular tachycardia requiring extensive resuscitative efforts. Intraoperative findings revealed biventricular failure and a clot in the right pulmonary artery. Despite aggressive treatment, including veno-arterial extracorporeal membrane oxygenation (VA ECMO), the patient's condition deteriorated, and life support was ultimately withdrawn. This case highlights the challenges of managing complex surgical patients and underscores the importance of multidisciplinary care in such cases.

## Introduction

Acute pulmonary embolism (PE) is an infrequent yet potentially life-threatening complication in the perioperative setting. The challenge of diagnosing PE is heightened in anesthetized patients, where conventional symptoms such as dyspnea, chest pain, hemoptysis, and syncope are not observable [1]. This highlights the importance of alternative indicators for timely diagnosis and intervention. Abrupt occurrence of unexplained tachycardia, hypotension, hypoxia, and decreased expired end-tidal carbon dioxide (ETCO_2_) levels may signal acute PE, necessitating prompt diagnostic exploration [1].

The progression of intraoperative PE differs from non-surgical cases where conscious patients respire spontaneously. Traditional approaches such as anticoagulation therapy are typically used to address venous thrombosis and PE in non-surgical situations. In the surgical patient, however, standard approaches become intricate challenges in the management of PE [1]. Contrasting trends in increased PaCO_2_ and decreased ETCO_2_ levels can serve as early indicators of potential PE in patients undergoing general anesthesia and mechanical ventilation [2].

Differential diagnosis of sudden cardiovascular collapse under general anesthesia includes allergic reactions, hemorrhage, hypovolemia, or pneumothorax. Complex cardiovascular events, including myocardial infarction, cardiac tamponade, or massive pulmonary embolism, must be considered. Intraoperative transthoracic echocardiography (TTE) has emerged as a preferred diagnostic tool, particularly in cases of hemodynamic collapse, allowing for diagnosis without interrupting ongoing surgical procedures [1]. Additionally, transesophageal echocardiogram (TEE) aids in directly visualizing PE within pulmonary arteries, enhancing the diagnosis of critical conditions.

For hemodynamically unstable patients, extracorporeal membrane oxygenation (ECMO) serves as a bridge to definitive interventions. ECMO helps stabilize compromised hemodynamics in high-risk PE cases featuring acute right ventricular failure, refractory hypoxia, and cardiac arrest [1]. Despite various reported treatment modalities, including anticoagulants, thrombolytic therapies, and surgical embolectomy, addressing circulatory collapse remains complex. The venoarterial-extracorporeal membrane oxygenation configuration (VA-ECMO) proves valuable as a bridging therapy to surgical embolectomy, endovascular approaches, or systemic thrombolysis [3].

## Case presentation

A 75-year-old female with a complex medical history was admitted to the hospital for an elective sigmoidectomy with ileorectal anastomosis following an inpatient admission, where she presented abdominal pain and vomiting.

The day before surgery, metoprolol was held because of low systolic blood pressure, but subsequently, the patient developed atrial fibrillation (AFib) with rapid ventricular response (RVR) with a heart rate in the 150s accompanied by dyspnea. AFib was controlled after the administration of three doses of 5mg IV metoprolol and the heart rate became stable in the 80s. Although the EKG displayed ST depression in V4 and V5, it was attributed to demand ischemia in the context of AFib with RVR, with normal troponin levels.

Before her transfer from an outside hospital (OSH), she received 5,000 units of subcutaneous heparin every 12 hours. In the operating room, her preoperative vital signs were stable with a blood pressure of 129/88, a pulse of 97 and a saturation of 92% on room air. However, upon induction with propofol 100 mg and rocuronium 10 mg, the patient immediately developed pulseless ventricular tachycardia. Endotracheal intubation was performed without difficulties. Extensive resuscitative efforts were carried out involving multiple rounds of CPR, defibrillation, and several doses of intravenous epinephrine. The patient experienced recurrent pulseless electrical activity (PEA) with subsequent return of spontaneous circulation (ROSC) eight times throughout the event. Persistent severe hypoxia, with oxygen saturation in the 60s, led to the initiation of advanced supportive measures, including invasive vascular access, with arterial line placement and central line insertion.

**Figure 1:**
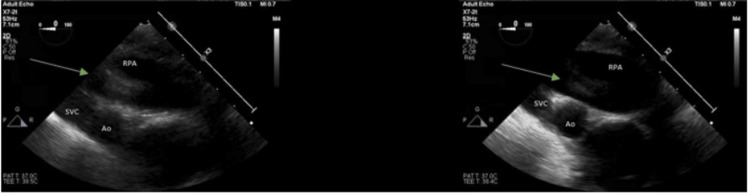
Upper esophageal ascending aorta view. A transesophageal echocardiogram was performed intraoperatively status post cardiac arrest, revealing biventricular systolic dysfunction, severe septal wall hypokinesis, 3+ mitral and tricuspid regurgitation, dilated left and right atria, and left ventricle ejection fraction of 30 ± 5%. A suspected clot in the right pulmonary artery (green arrow).

During cardiac arrest, TEE revealed biventricular failure with a left ventricular ejection fraction (LVEF) of 30% (See Figure 1) and a clot in the right pulmonary artery. Due to the patient's critical hemodynamic instability and the need for maximum ventilatory and vasopressor support, emergent aspiration pulmonary thrombectomy was attempted but was unsuccessful. This raised concerns about potential distal embolization.

The surgery was canceled, and the patient was transferred to the Cardiovascular Intensive Care Unit (CICU) on 100% FiO2. After resuscitation, arterial blood gas (ABG) revealed severe metabolic acidosis (pH 7.09) and hypoxia (PaO_2_ 65) which prompted the initiation of VA-ECMO. Despite transfusions and critical care support, the patient's condition remained compromised, marked by persistent metabolic acidosis and hypoxia.

**Figure 2:**
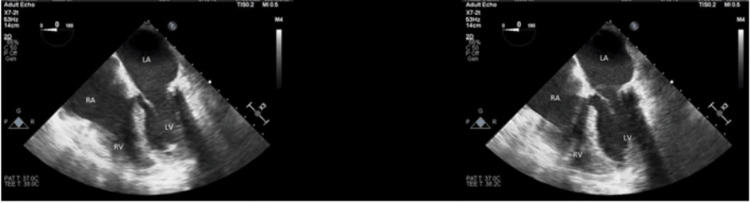
Mid-esophageal four-chamber view. A transesophageal echocardiogram was performed six hours after cardiac arrest, revealing moderately dilated right and left ventricles, biventricular systolic dysfunction, +1 mitral regurgitation, and akinetic septal wall. Left ventricular systolic function is severely reduced with an ejection fraction of 10 ± 5%.

Myocardial infarction (MI) versus PE was considered as a potential cause for the recurrent PEA (See Figure 2), but a downtrend in high sensitivity troponin T (TnT) made this differential less likely. The echocardiogram displayed the absence of pericardial effusion therefore excluding tamponade, and the chest X-ray was not indicative of pneumothorax. Unfortunately, the patient's neurological condition continued to deteriorate until examination demonstrated absence of reflexes. In consultation with the Health Care Power of Attorney (HCPOA), the decision was made to withdraw life support, ultimately resulting in the patient's passing two days after the acute cardiopulmonary event.

## Discussion

PE in a surgical patient should be considered when patients show signs of perioperative hypoxemia, hypotension, tachycardia, or a sudden decrease in end-tidal CO2. Perioperative PE can lead to rapid and potentially fatal hemodynamic instability. When evaluating a patient for PEA following anesthesia induction, it is essential to consider multiple potential differentials [4]. While acute PE emerges as the most likely cause, alternative diagnoses such as myocardial infarction (MI), cardiac tamponade, pneumothorax, and medication side effects warrant thorough investigation.

A critical aspect of the differential diagnosis is the consideration of myocardial infarction (MI). The diagnostic criteria for MI involve a discernible rise and fall of cardiac troponin, with at least one value surpassing the 99th percentile of a healthy reference population, coupled with signs or symptoms of ischemia [5]. In contrast, patients with a PE typically exhibit sustained troponin levels above the 99th percentile [5]. In this case, the absence of the typical troponin changes, coupled with pre-existing biventricular systolic dysfunction and septal Q waves on electrocardiogram, diminishes the likelihood of an MI diagnosis.

Cardiac tamponade is associated with acute decompensation and potential cardiac arrest and therefore should also be considered. A clinical diagnosis of tamponade necessitates a hemodynamically unstable patient with pericardial effusion [6]. Given the patient's history of coronary artery disease (CAD), a ventricular free wall rupture leading to fluid accumulation in the pericardial space could mimic cardiac tamponade. The similarity in presentation, with fluid hindering diastolic relaxation causing hypotension and eventual pulseless electrical activity, was contemplated [6]. Nevertheless, ruling out this possibility occurred during the intraoperative echocardiogram, which did not reveal any visualized pericardial fluid.

The initial clinical manifestations in this instance also raised the possibility of a pneumothorax; however, no corroborating evidence was found. The central venous catheterization procedure, a potential cause of pneumothorax, was conducted under ultrasound guidance to minimize this risk and resulted without discernible abnormalities. Moreover, real-time intraoperative auscultation affirmed bilateral breath sounds, and a chest X-ray (CXR) subsequently ruled out pneumothorax.

The patient had a documented history of Afib, managed with the direct oral anticoagulant (DOAC) rivaroxaban. In this case, the patient took her last dose of rivaroxaban four days before her sigmoidectomy and was bridged immediately to heparin when she was transferred from OSH. Cessation of anticoagulation in the perioperative period may have posed an elevated risk of thromboembolic events, given that surgical procedures inherently induce a hypercoagulable state [7].

The cause of this patient’s PEA following induction was confirmed to be a PE, substantiated by the TEE evidencing the clot in the pulmonary artery that contributed to the ventricular dysfunction. Intraoperatively, the patient exhibited acute hemodynamic alterations consistent with PE presentation, including tachycardia, hypotension, hypoxemia, and a decrease in ETCO_2_ [1]. The intraoperative echocardiogram also disclosed biventricular hypokinesis, a set of findings indicative of a potential PE [8]. The TEE played a pivotal role in this case, as it further confirmed suspicion of a PE [9].

## Conclusions

The occurrence of perioperative PE can result in rapid and potentially life-threatening hemodynamic instability. Swift therapeutic decision-making is crucial, requiring a comprehensive understanding of risk factors, diagnostic tools, and a multimodal approach to risk stratification and management. While extracorporeal membrane oxygenation (ECMO) is not a universal solution, its prompt application may improve patient prognoses if hemodynamic collapse persists after initial treatment. This case highlights the challenges in diagnosing and managing perioperative complications, emphasizing the importance of vigilance, adaptability in decision-making, and the potential of advanced imaging techniques in challenging scenarios.
